# Supplementary material for: MetaRibo-Seq measures translation in microbiomes
Source: Nat Commun. 2020 Jun 29;11:3268. doi: 10.1038/s41467-020-17081-z (PMC7324362; doi:10.1038/s41467-020-17081-z)
Supplement: Supplementary file 10 — Supplementary Data 7 [file 41467_2020_17081_MOESM10_ESM.zip › File2/Confidence_High_Taxonomy/220634_out.krona.html]

Javascript must be enabled to view this page.

members
magnitude
magnitudeUnassigned
count
unassigned
taxon
rank

220634\_out

190

190
superkingdom
2

190
976
phylum

190
200643
class

order
171549
190

1
2005519
family

1
genus
1348911

1
species

SRS014235\_contig\_number\_46098
1941478

189
815
family

189
genus
816

1

SRS1055022\_contig\_number\_9943
291645
species


SRS011084\_contig\_number\_21653SRS056273\_contig\_number\_13493
1262750
species
2

1

SRS014948\_contig\_number\_22544
329854
species

species

SRS021484\_contig\_number\_13015
1263037
1

818

SRS020233\_contig\_number\_41791SRS1041139\_contig\_number\_1944SRS146813\_contig\_number\_26331
species
3

2
species

SRS019685\_contig\_number\_16338SRS893252\_contig\_number\_9029
338188

29523

SRS058770\_contig\_number\_11388
species
1

357276

SRS013098\_contig\_number\_42734
species
1

3

SRS024331\_contig\_number\_11485SRS043768\_contig\_number\_16192SRS077849\_contig\_number\_2006
46506
species

172

SRS011134\_contig\_number\_46365SRS011302\_contig\_number\_23202SRS012273\_contig\_number\_49597SRS012849\_contig\_number\_contig-100\_1997.207408SRS012969\_contig\_number\_33509SRS013476\_contig\_number\_27944SRS013521\_contig\_number\_12401SRS013687\_contig\_number\_492SRS013951\_contig\_number\_contig-100\_7349.179756SRS013965\_contig\_number\_16147SRS014287\_contig\_number\_10157SRS014313\_contig\_number\_1962SRS014412\_contig\_number\_contig-100\_1640.120332SRS014613\_contig\_number\_13862SRS014923\_contig\_number\_35797SRS014979\_contig\_number\_5657SRS015264\_contig\_number\_4536SRS015663\_contig\_number\_15462SRS015694\_contig\_number\_19787SRS015960\_contig\_number\_3752SRS016018\_contig\_number\_12472SRS016203\_contig\_number\_5601SRS016335\_contig\_number\_13680SRS016541\_contig\_number\_2150SRS016954\_contig\_number\_22404SRS016989\_contig\_number\_10101SRS017103\_contig\_number\_4858SRS017191\_contig\_number\_4487SRS017701\_contig\_number\_706SRS017821\_contig\_number\_29335SRS018133\_contig\_number\_1869SRS018313\_contig\_number\_10925SRS018427\_contig\_number\_contig-100\_1203.187367SRS018541\_contig\_number\_2315SRS018888\_contig\_number\_10265SRS019161\_contig\_number\_2801SRS019178\_contig\_number\_1669SRS019267\_contig\_number\_contig-100\_164.57629SRS019381\_contig\_number\_10859SRS019397\_contig\_number\_2946SRS019601\_contig\_number\_15282SRS019910\_contig\_number\_24621SRS020271\_contig\_number\_1293SRS020328\_contig\_number\_26392SRS020394\_contig\_number\_3900SRS021219\_contig\_number\_880SRS022071\_contig\_number\_24060SRS022137\_contig\_number\_20559SRS023526\_contig\_number\_1617SRS023583\_contig\_number\_3176SRS023715\_contig\_number\_4488SRS023914\_contig\_number\_1697SRS024492\_contig\_number\_4752SRS024625\_contig\_number\_9609SRS042966\_contig\_number\_19534SRS043001\_contig\_number\_4537SRS043667\_contig\_number\_4671SRS043701\_contig\_number\_11129SRS043841\_contig\_number\_3985SRS045004\_contig\_number\_1668SRS045244\_contig\_number\_1294SRS045826\_contig\_number\_3758SRS046068\_contig\_number\_412SRS047014\_contig\_number\_23028SRS047044\_contig\_number\_17022SRS047433\_contig\_number\_17529SRS048060\_contig\_number\_3911SRS048164\_contig\_number\_11123SRS049712\_contig\_number\_3731SRS049773\_contig\_number\_6469SRS050422\_contig\_number\_24059SRS050752\_contig\_number\_11527SRS050925\_contig\_number\_2668SRS051031\_contig\_number\_10253SRS051882\_contig\_number\_3365SRS052697\_contig\_number\_16937SRS053398\_contig\_number\_10611SRS053573\_contig\_number\_275SRS053649\_contig\_number\_20395SRS054352\_contig\_number\_15220SRS054905\_contig\_number\_9479SRS056259\_contig\_number\_13240SRS058723\_contig\_number\_6035SRS062427\_contig\_number\_7505SRS062654\_contig\_number\_3902SRS063370\_contig\_number\_1354SRS064276\_contig\_number\_21301SRS064557\_contig\_number\_9261SRS064973\_contig\_number\_2442SRS065397\_contig\_number\_13223SRS065504\_contig\_number\_1799SRS074964\_contig\_number\_458SRS075821\_contig\_number\_3081SRS075878\_contig\_number\_2693SRS076929\_contig\_number\_contig-100\_3677.124752SRS077024\_contig\_number\_11788SRS077086\_contig\_number\_3061SRS077294\_contig\_number\_13625SRS077392\_contig\_number\_32471SRS077454\_contig\_number\_4417SRS077502\_contig\_number\_3351SRS077589\_contig\_number\_4147SRS078176\_contig\_number\_12623SRS078242\_contig\_number\_21495SRS098571\_contig\_number\_1464SRS098644\_contig\_number\_33840SRS098827\_contig\_number\_1000SRS100907\_contig\_number\_88SRS101376\_contig\_number\_19326SRS103987\_contig\_number\_8252SRS1041031\_contig\_number\_10784SRS1041039\_contig\_number\_9960SRS1041091\_contig\_number\_17209SRS1041092\_contig\_number\_14787SRS1041118\_contig\_number\_3030SRS1041133\_contig\_number\_2925SRS1041134\_contig\_number\_6458SRS1041137\_contig\_number\_2893SRS1041138\_contig\_number\_13301SRS1041141\_contig\_number\_contig-100\_135.83174SRS1041145\_contig\_number\_contig-100\_2765.81919SRS1041157\_contig\_number\_4583SRS104400\_contig\_number\_24930SRS104975\_contig\_number\_18787SRS105082\_contig\_number\_4023SRS105153\_contig\_number\_1845SRS1054716\_contig\_number\_15481SRS1055049\_contig\_number\_3804SRS1055076\_contig\_number\_11137SRS142542\_contig\_number\_1405SRS142599\_contig\_number\_24742SRS142781\_contig\_number\_1634SRS142890\_contig\_number\_8351SRS142923\_contig\_number\_19940SRS143342\_contig\_number\_10566SRS143523\_contig\_number\_23464SRS143598\_contig\_number\_21298SRS143598\_contig\_number\_contig-100\_3797.46081SRS143722\_contig\_number\_3842SRS143876\_contig\_number\_contig-100\_1966.160992SRS143895\_contig\_number\_38309SRS144183\_contig\_number\_1553SRS144603\_contig\_number\_contig-100\_987.104621SRS145308\_contig\_number\_contig-100\_4554.4555SRS145497\_contig\_number\_9103SRS147039\_contig\_number\_2952SRS147346\_contig\_number\_9436SRS147425\_contig\_number\_3168SRS147614\_contig\_number\_8174SRS147977\_contig\_number\_1784SRS148091\_contig\_number\_12265SRS148319\_contig\_number\_9011SRS148424\_contig\_number\_904SRS148721\_contig\_number\_43343SRS148817\_contig\_number\_1681SRS149181\_contig\_number\_22383SRS149244\_contig\_number\_431SRS149784\_contig\_number\_27100SRS149879\_contig\_number\_23461SRS150029\_contig\_number\_6264SRS892882\_contig\_number\_13033SRS893279\_contig\_number\_13534SRS893285\_contig\_number\_40SRS893292\_contig\_number\_7292SRS893295\_contig\_number\_10831SRS893300\_contig\_number\_2112SRS893327\_contig\_number\_4590SRS893366\_contig\_number\_9506SRS893373\_contig\_number\_contig-100\_4210.111112SRS893378\_contig\_number\_11185SRS893382\_contig\_number\_5091SRS971276\_contig\_number\_2695
47678
species

species

SRS075984\_contig\_number\_12646
821
1

species
204516

SRS045645\_contig\_number\_569
1
